# Semiconductor quantum dot-sensitized solar cells

**DOI:** 10.3402/nano.v4i0.22578

**Published:** 2013-10-31

**Authors:** Jianjun Tian, Guozhong Cao

**Affiliations:** 1 Advanced Materials and Technology Institute, University of Science and Technology Beijing, Beijing, China; 2 Department of Materials and Engineering, University of Washington, Seattle, WA, USA

**Keywords:** quantum dot, solar cell, quantum dot–sensitized solar cell (QDSC), quantum confinement, multiple exciton generation (MEG), photoelectrode

## Abstract

Semiconductor quantum dots (QDs) have been drawing great attention recently as a material for solar energy conversion due to their versatile optical and electrical properties. The QD-sensitized solar cell (QDSC) is one of the burgeoning semiconductor QD solar cells that shows promising developments for the next generation of solar cells. This article focuses on recent developments in QDSCs, including 1) the effect of quantum confinement on QDSCs, 2) the multiple exciton generation (MEG) of QDs, 3) fabrication methods of QDs, and 4) nanocrystalline photoelectrodes for solar cells. We also make suggestions for future research on QDSCs. Although the efficiency of QDSCs is still low, we think there will be major breakthroughs in developing QDSCs in the future.

The establishment of low-cost and high-performance solar cells for sustainable energy sources to replace fossil fuels has become an urgent subject to scientists around the world (1, 2). Because traditional photovoltaic devices (i.e. the p-n junction silicon crystalline solar cells) suffer from high costs of manufacturing and installation, now the focus is on the next generation of solar cells with high efficiency at economically viable costs. As a cost-effective alternative to silicon-based photovoltaics, semiconductor quantum dot (QD)-sensitized solar cells (QDSCs) have attracted considerable attention recently and have shown promising developments for the next generation of solar cells (2–7). QDSCs can be regarded as a derivative of dye-sensitized solar cells (DSCs), which were first reported by O'Regan and Grätzel in 1991 (8). In DSCs, the sensitizer commonly uses organic dyes of ruthenium polypyridine complexes. To increase the light harvest in the visible light region, many efforts have been made to focus on the development of high-performance sensitizers (9–12). It has always been a challenge to obtain an ideal organic dye as a sensitizer to absorb photons in the full sunlight spectra. For this reason, narrow-band-gap semiconductor QDs, such as CdS (13, 14), CdSe (15, 16), PbS (17), and InAs (18), have been used as the photosensitizer instead of organic dyes due to their versatile optical and electrical properties (19–22), including: 1) a tunable band gap depending on the QD size, 2) a larger extinction coefficient, 3) higher stability toward water and oxygen, and 4) multiple exciton generation (MEG) with single-photon absorption (23–25). The theoretical photovoltaic conversion efficiency of QDSCs can reach up to 42% in view of the MEG effect of QDs. Such efficiency is much higher than the rate of 31% for semiconductor solar cells according to the Schockley–Queisser limit (26).

Figure 1(a) shows the cell structure of a QDSC, which consists of a wide-band-gap mesoporous oxide film (a photoelectrode, such as the commonly used TiO_2_ or ZnO), QDs (the sensitizer), an electrolyte, and a counter-electrode. During operation, photons are captured by QDs, yielding electron–hole pairs that are rapidly separated into electrons and holes at the interface between the nanocrystalline oxide and QDs. The electrons jump into the oxide film, and the holes are released by redox couples in the electrolyte. Figure 1(b) shows photoinduced charge transfer processes employing S^2−^/Sn^2−^ as the redox couple (7): 1) charge injection from an excited QD into TiO_2_, 2) transport of electrons to the collecting electrode surface, 3) hole transfer to the redox couple, 4) regeneration of the redox couple, 5) recombination of electrons from the QD and the oxidized form of the redox couple, and 6) interfacial recombination of electrons from TiO_2_ and the oxidized form of the redox couple. Kamat group's works reported (27, 28) that the electron transfer between QD and TiO_2_ was an ultrafast process with a rate constant of the order of 10^10^∼10^11^ s^−1^, which was faster than that of hole transfer (10^7^∼10^9^ s^−1^). However, electron transport within the mesoporous TiO_2_ film is slower than that of electron and hole transfers. So the recombination losses become a major factor in limiting the overall efficiency.

**Fig. 1:**
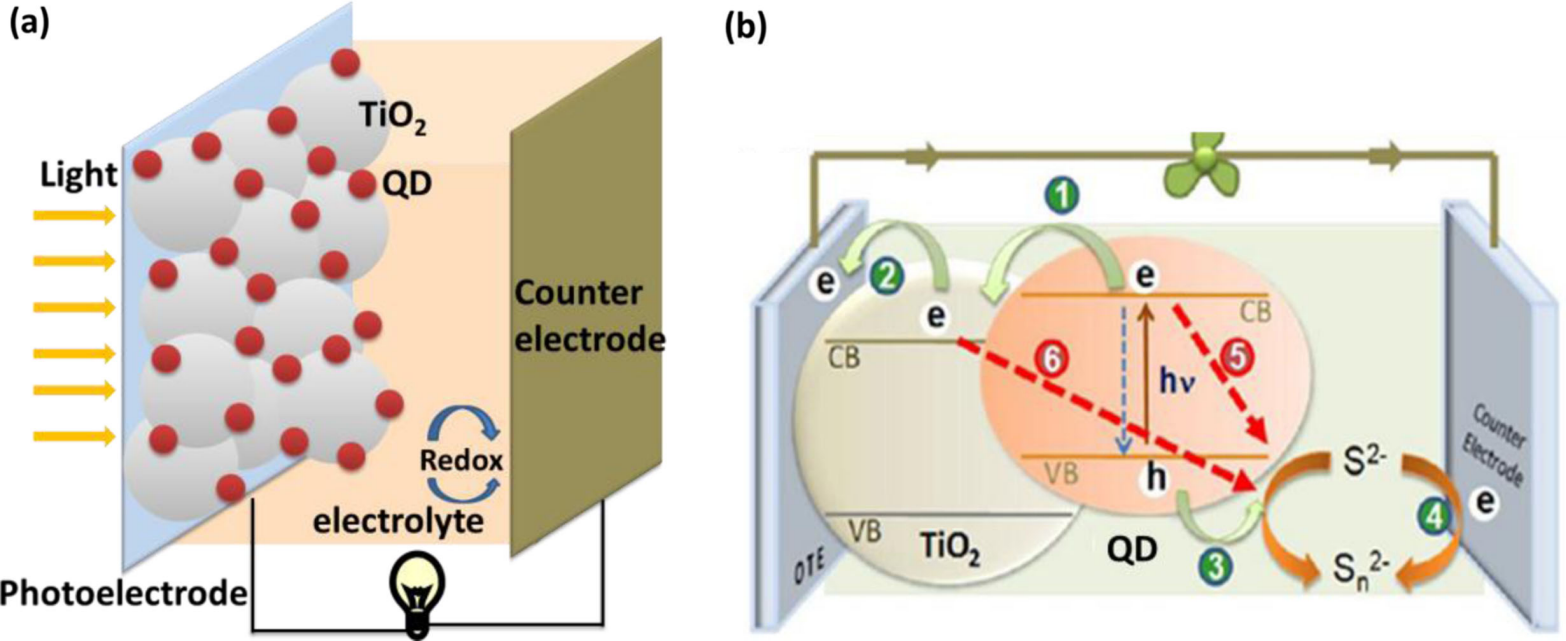
(a) Schematic illustration of the structure of a quantum dot–sensitized solar cell (QDSC); and (b) schematic illustration of photoinduced charge transfer processes following a laser pulse excitation (7).

## Quantum confinement effect for QDSCs

QDs are extremely small semiconductor nanocrystals with a size comparable to the Bohr radius of an exciton (29). For most semiconductors, the Bohr radius of an exciton is in the range of 1∼10 nm: for example, it is 4.2 nm for Si, 3.1 nm for CdS, 6.1 nm for CdSe, and 2.2 nm for ZnO. However, the Bohr radius of the exciton is very large for some semiconductors: it is 20.4 nm for PbS, 46 nm for PbSe, and 67.5 nm for InSb. Due to the dimension effect, the behavior of electrons in QDs differs from that in the corresponding bulk material, which is called the ‘quantum confinement effect’. A semiconductor with a larger excitonic Bohr radius means that the QDs made from the material may achieve a strong confinement effect more easily. Because of the quantum confinement effect, the band gap energy (*E*_*g*_) of QD increases with the decrease of particle size (30–32). *E*_*g*_ can be elucidated by *E*_*g*_ ∝ 1/*r* ^2^, where *r* is the radius of QD (29). The increase of *E*_g_ means that more energy will be needed in order to be absorbed by the QD. So the range of optical absorption wavelengths of QD can be tuned by controlling the size of QD. Such a feature of QDs with tunable *E*_*g*_ has led to their applications in light-emitting diodes (LEDs) for full-color displays (33), and in QD-sensitized solar cells for the generation of optical absorption at desired wavelengths (34, 35).

**Fig. 2:**
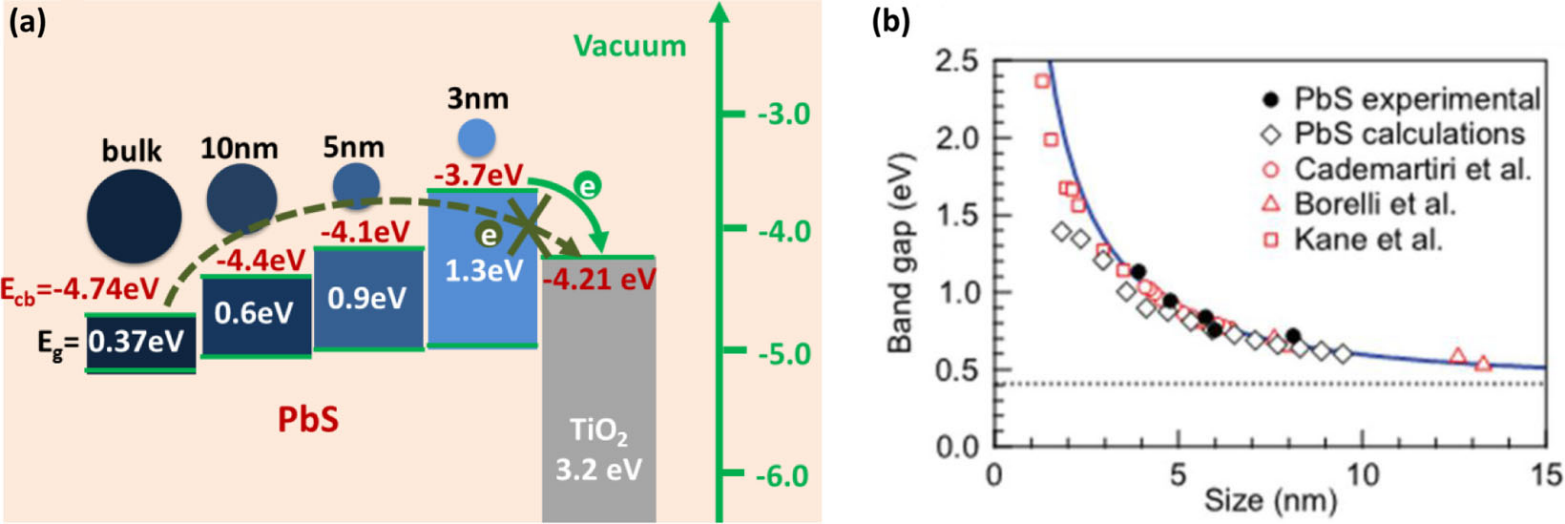
(a) Schematic illustration of the modulation of energy levels of PbS by particle size; and (b) relationship between the PbS *E*_g_ and particle size, as reported in Refs. (31, 32).

**Fig. 3:**
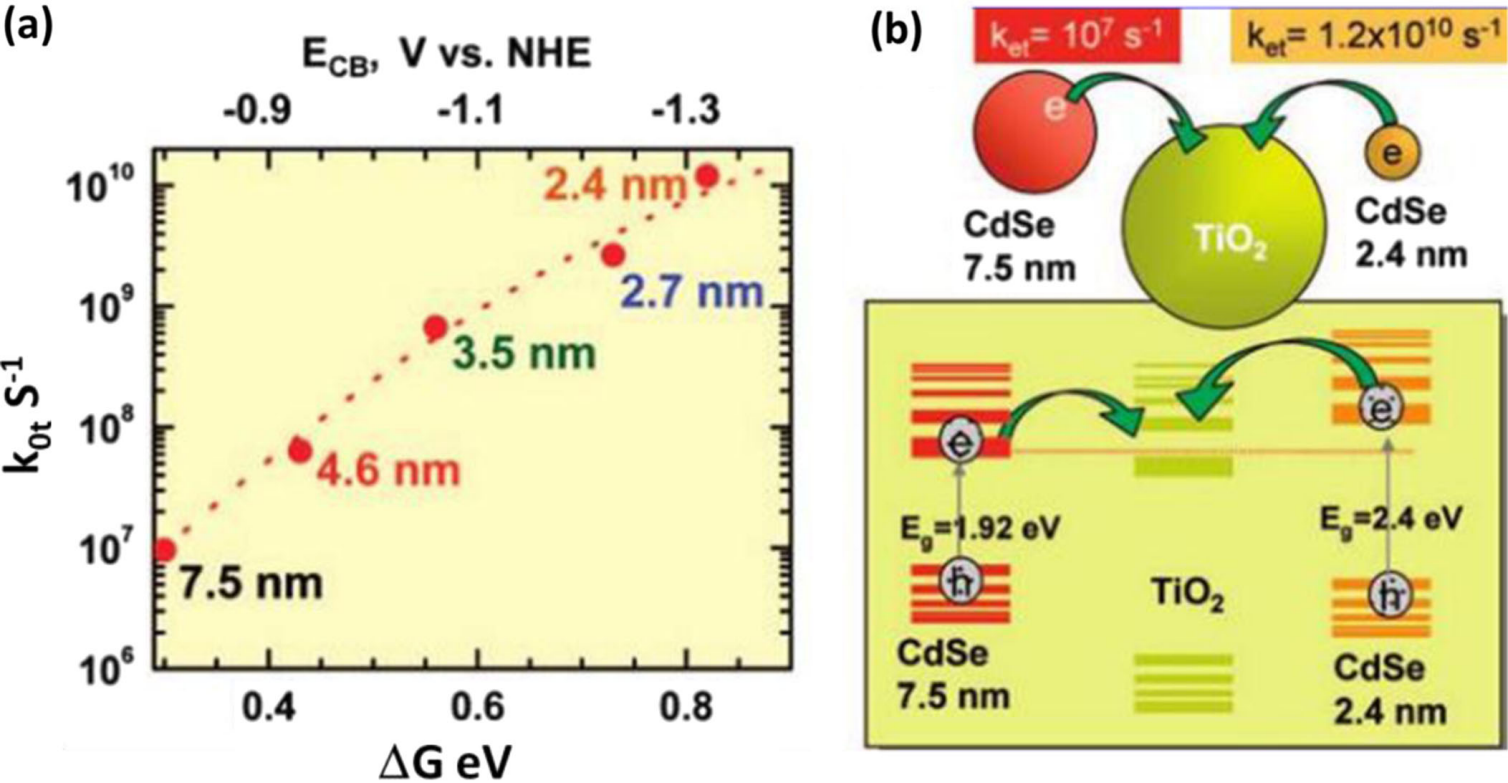
(a) The dependence of the electron transfer rate constant on the energy difference between the conduction bands; and (b) a scheme illustrating the principle of electron transfer from two different-sized CdSe quantum dots into a TiO_2_ nanoparticle (26).

**Fig. 4:**
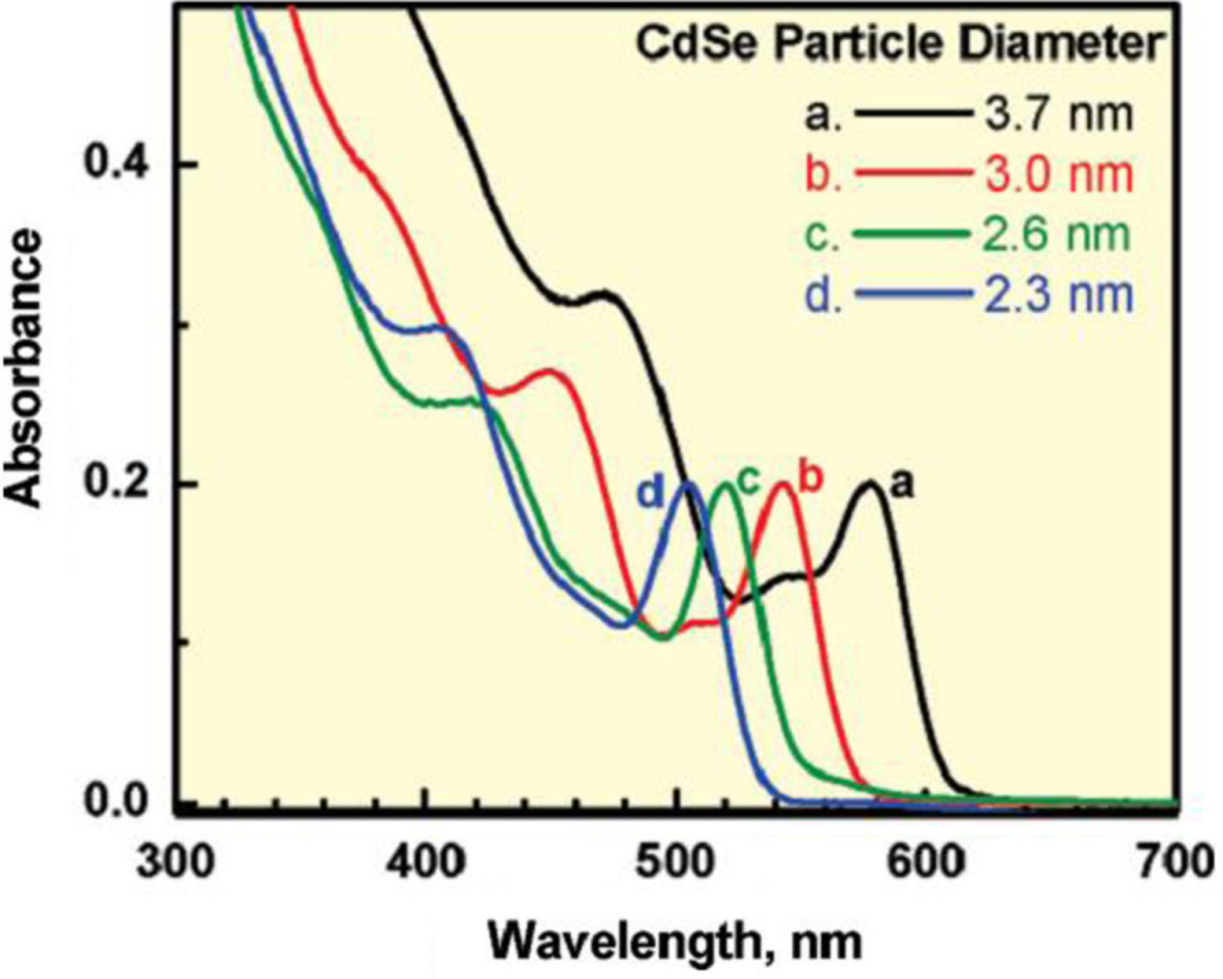
Absorption spectra of 3.7, 3.0, 2.6, and 2.3 nm diameter CdSe quantum dots in toluene; from Ref. (37).

Ref. (36) has demonstrated that the conduction band energy (*E*_cb_) of QD would move up the high energy level by decreasing particle size due to the quantum confinement effect, which is very significant to solar cells. As shown in Fig. 2(a), the *E*_cb_ of bulk PbS is −4.74 eV, which is lower than that of TiO_2_ (−4.21 eV). The electrons in the conduction band of bulk PbS is difficult to jump into the conduction band of TiO_2_. The *E*_g_ of PbS can be increased by reducing its particle size, as shown in Fig. 2(b), leading to the conduction band minimum shift to higher energy. When the conduction band energy of PbS matches that of TiO_2_, the electrons divided from exciton pairs by photons can easily transfer from the conduction band in PbS into the conduction band in TiO_2_. So the quantum confinement effect is the necessary condition for the construction of QDSCs. For QDSCs, smaller QDs are preferred in order to possibly achieve more QDs adsorbed on the photoelectrode film. Smaller QDs have also demonstrated a higher electron injection rate than their larger counterparts. Figure 3 shows the dependence of the electron transfer rate constant on the energy difference between the conduction bands and the principle of electron transfer from two different-sized CdSe QDs into a TiO_2_ nanoparticle (26). It can be seen that the electron transfer rate evidently increases with decreasing QD size in the CdSe–TiO_2_ system. For QDs, the increase of band gap would be expected to have favorable conduction band energies for injecting electrons into a photoelectrode. However, the increase of *E*_g_ indicates that only high energy photons can be absorbed by the QD, leading to the absorption wavelength edge of the QD blue shift as shown in Fig. 4. Too small QDs will lead to too much low the optical absorption for the photoelectrodes, which has negative impacts on the solar cells. Therefore, the best solar-to-electricity conversion efficiency can be obtained by optimizing the band energy structure of QDs to match the oxide film and obtain a wide optical absorption wavelength.

Table 1 shows the energy band parameters of QDs and oxides, which are usually chosen to fabricate QDSCs. The principles for choosing materials to construct solar cells are as follows: 1) optical absorption is primarily determined by the band gap of active materials, and therefore the materials with narrow and direct band gaps are preferred; and 2) the device structure should be designed by choosing materials with well-matched energy levels that may establish a suitable energy gradient, allowing the charges to transport highly efficiently within the solar cell (29).

**Table 1:** Energy band parameters of some of the most commonly used materials for QDSCs (38, 39)

Semiconductors	Band gap (eV)	Conduction band minimum (eV)	Valence band maximum (eV)
CdS	2.40	−3.98	−6.38
CdSe	1.74	−4.10	−5.84
PbS	0.37	−4.74	−5.11
PbSe	0.27	−4.93	−5.20
ZnS	3.60	−3.46	−7.06
ZnSe	2.70	−3.40	−6.10
CuInS_2_	1.50	−4.06	−5.56
TiO_2_	3.20	−4.21	−7.41
ZnO	3.20	−4.19	−7.39
SnO_2_	3.50	−4.50	−8.00

## The MEG effect for QD-sensitized solar cells

MEG in QDs has been considered another way to enhance the power conversion efficiency of QDSCs by utilizing the excess energy in the absorbed photons. The MEG effect is that two or more electron–hole pairs (excitons) are generated by one photon excitation, in contrast with the conventional case where one photon excitation can produce only a single exciton. In theory, the MEG effect requires a photon with energy at least twice that of the band gap of the QDs. In view of the MEG effect, the theoretic power conversion efficiency of QDSC has been predicted to be as high as 42%, which is higher than the Shockley–Queisser efficiency limit of 31%, for the traditional single-junction solar cells (40). The experimental study of Semonin (41) has proven the feasibility of this concept, with the demonstration of an external quantum efficiency greater than 100% at wavelengths below 400 nm on a p-n junction solar cell based on a layer of PbSe QDs deposited on ZnO thin film. So the utilization of high-energy photons to generate multiple excitons or capture hot electrons before their thermalization can boost the operational efficiency of QDSC (7).

**Fig. 5:**
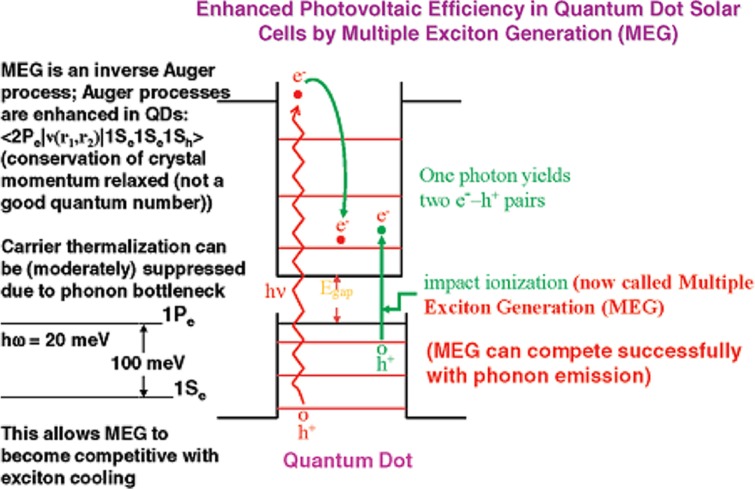
Multiple exciton generation in quantum dots (40).

The MEG effect is a phenomenon that can also be observed in a bulk semiconductor. However, the required threshold for the energy of photons is much higher than that in QDs. For the semiconductor PbSe, the threshold energy of bulk material is as high as 6.5 *E*_g_, whereas it is about 3.4 *E*_g_ of PbSe QD (*E*_g_ is the energy band gap of the PbSe). The possibility of enhanced MEG in QDs was first proposed in 2001, and the original concept is shown in Fig. 5, (40). The possible reasons for the MEG effect, which can be achieved easily in QDs, have been attributed to the following (40): 1) the electron–hole (e^−^–h^+^) pairs are correlated and thus exist as excitons rather than free carriers; 2) the rate of hot electron and hole cooling can be slowed because of the formation of discrete electronic states; 3) momentum is not a good quantum number, and thus the need to conserve crystal momentum is relaxed; and 4) auger processes are greatly enhanced because of the increased e^−^–h^+^ Coulomb interaction. So the production of multiple exciton pairs in QDs can be enhanced in comparison with bulk semiconductors.

MEG in QDs is a very important process that, if harnessed, can lead to a new solar conversion efficiency limit (42). Although reasonably high and reliable MEG efficiencies have now been reported, no QD-based solar cells have yet shown enhanced conversion efficiency due to MEG effects (40). So the achievement of QD solar cells with MEG effect enhancement is still facing a huge challenge, partially because the excitation of the MEG effect requires photons with relatively high energy and a pump light with high power density (29). To make the largest impact on solar energy technologies, the MEG efficiency needs to be further improved so that the onset of MEG occurs as close to 2 *E*_g_ as possible (42).

## Fabrication methods of QDs for the solar cells

**Fig. 6:**
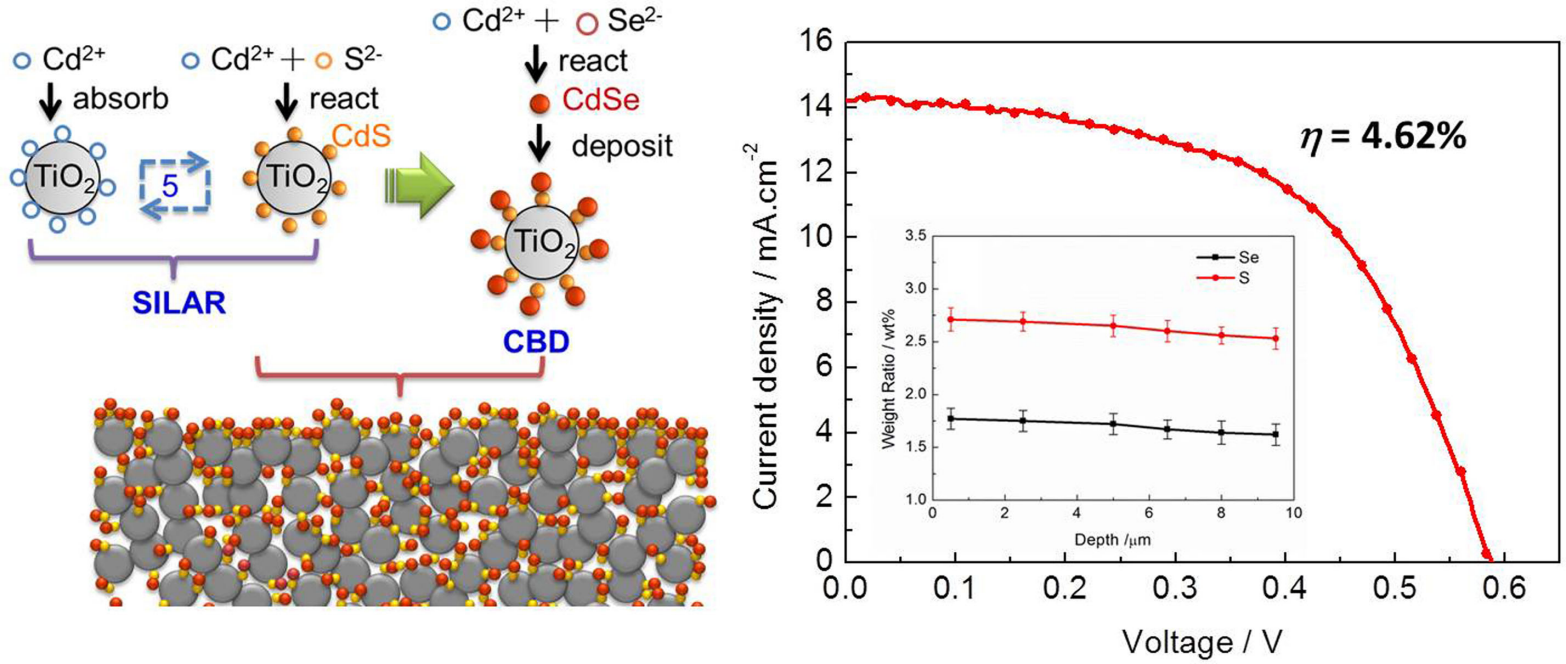
Sketch of the formation of CdS–CdSe QDs on mesoporous TiO_2_ film and the J–V curve of QDSCs.

In a typical process for the fabrication of QDSC photoelectrodes, QDs can be introduced via two approaches: 1) in situ growth directly from precursor solutions, and 2) adsorption of pre-synthesized QDs with or without a bifunctional linker. However, the QDSCs produced by the latter approach have relatively low conversion efficiency, largely due to the difficulty in achieving sufficient coverage of QDs (15). The former (i.e. in situ growth of QDs) includes chemical bath deposition (CBD) (43) and successive ionic layer absorption and reaction (SILAR) (24, 44), and it has been shown to perform better than the latter when being adopted to assemble QDSCs (45). The CBD is a relatively simple method to deposit QDs and nanoparticle films, and it possesses many advantages, such as stable yieldings, robust adherence, and uniform and good reproducibility. The growth of QDs strongly depends on the growth conditions, such as the duration of deposition, composition and temperature of the solution, and characteristics of the mesoporous films. The SILAR method is based on successive reactions on the surface oxides. Each reaction is followed by rinsing, which enables a heterogeneous reaction between the solid phase and the solvated ions in the solution. So a thin film can be grown layer by layer. Figure 6 shows the schematic illustration of the formation process of CdS–CdSe QD co-sensitized solar cells. The CdS and CdSe on a TiO_2_ mesoporous film are synthesized by the SILAR and CBD methods, respectively. The QDSCs assembled with a TiO_2_ mesoporous film, CdS–CdSe QDs, a polysulfide electrolyte, and a Cu_2_S counter-electrode exhibit a high power conversion efficiency of 4.62% (25).

## Nanocrystalline photoelectrodes for solar cells

As wide-band-gap semiconductors for the sensitizer scaffold, conventional TiO_2_ and ZnO porous nanocrystalline films had been used in QDSCs due to the large surface area available for QD adsorption. TiO_2_ is commonly chosen as the photoelectrode of QDSCs, and it exhibits high power conversion efficiency (∼5%). For example, the research of Hossain et al. (46) showed that CdSe-sensitized TiO_2_ solar cells incorporating light-scattering layers presented a power conversion efficiency of 5.21%. Kamat et al. reported that QDSCs with Mn-doped CdS–CdSe on the TiO_2_ film achieved a power conversion efficiency of 5.4% (3). Lee et al. (47) developed a PbS–Hg QD-sensitized TiO_2_ solar cell with an unprecedentedly high power conversion efficiency of 5.6%.

**Fig. 7:**
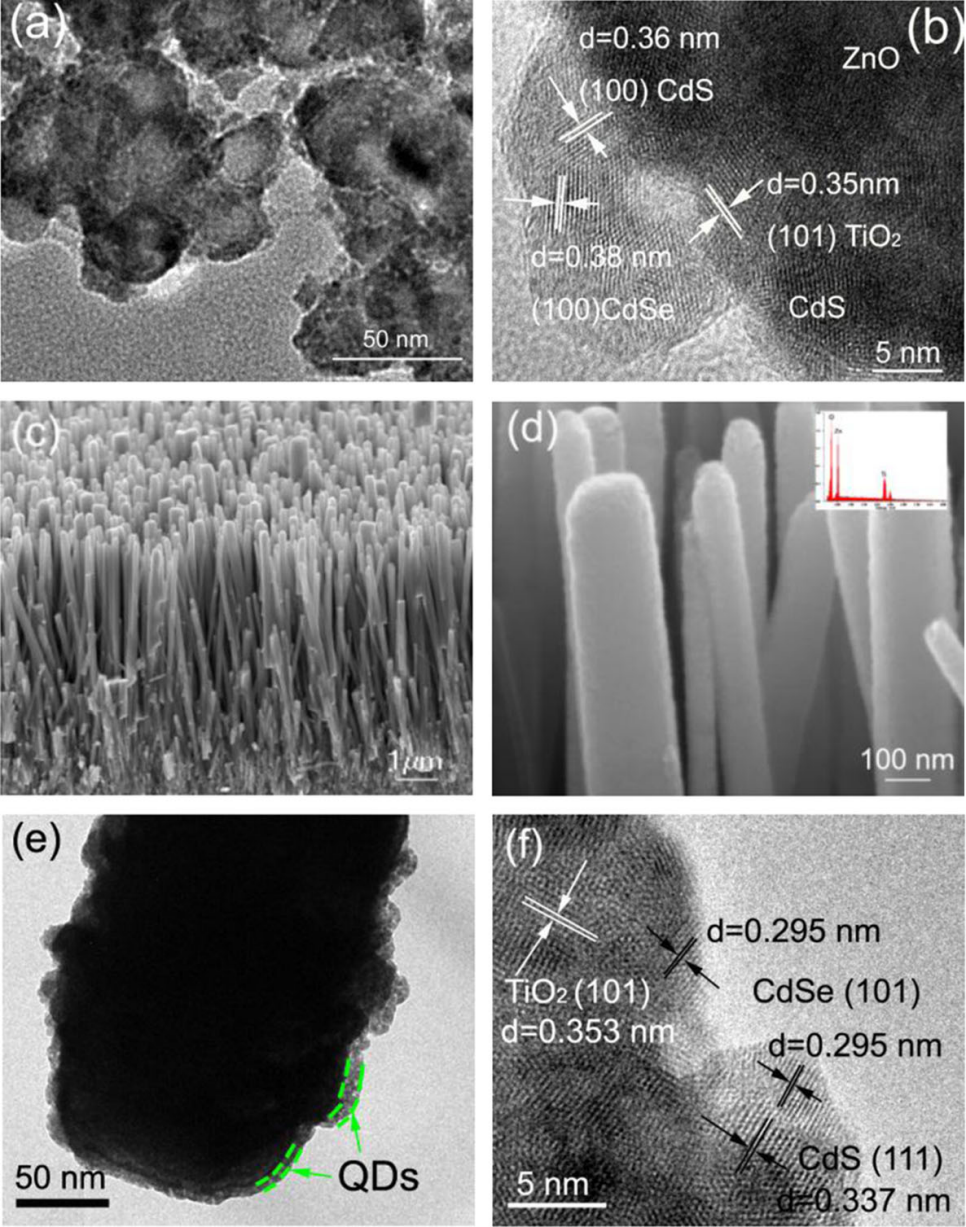
(a) Transmission electron microscopy (TEM) and (b) high-resolution TEM (HRTEM) images of passivated ZnO nanoparticles film loaded with QDs; (c) low- and (d) high-magnification scanning electron microscopy images of the passivated ZnO nanowires array (inset shows energy-dispersive spectroscopy spectra of the passivated arrays); and (e) TEM and (f) HRTEM images of a ZnO nanowires array loaded with QDs.

ZnO is a good alternative to TiO_2_ because it possesses energy-band structure and physical properties that are similar to those of TiO_2_. But it has an electronic mobility ∼4 times higher than that of TiO_2_ (48–50). In addition, ZnO is easy to form anisotropic structures (such as nanowires, nanorods, and nanotubes), which presents unique electronic and optical properties (51, 52). Furthermore, a photoelectrode film constructed with these nanostructures is helpful for the distribution of QDs (25). ZnO-nanostructured photoelectrodes for QDSCs have been investigated over the last several years (53–57). However, the efficiency of ZnO-based QDSCs is lower than that of TiO_2_-based devices, which is likely due to the high surface charge recombination in ZnO (58, 59). The high surface charge recombination can be attributed to many defects of the ZnO surface. In addition, the chemical stability of ZnO is less than that of TiO_2_, which makes it easy for ZnO to react with the electrolyte (60). Tian et al. (61) developed a facile passivation strategy for ZnO mesoporous photoelectrodes. This method not only opened the apertures to improve the distribution of QDs in the photoelectrodes, increased the specific surface area, and reduced the surface defects of the ZnO photoelectrodes to accommodate more QDs, but also suppressed the charge recombination and prolonged the electron lifetime by introducing a barrier layer. As a result, a record power conversion efficiency of 4.68% for ZnO-based QDSCs was obtained. Figure 7(a) and 7(b) show the transmission electron microscopy (TEM) and high-resolution TEM images of the passivated ZnO loaded with QDs, showing the passivated ZnO coated by TiO_2_ and CdS–CdSe QDs that are 4–6 nm in size. In addition, this passivation strategy can also be applied in ZnO nanowires to enhance their performance (as shown in Fig. 7(c)–(f)) (62).

## Summary and future work

Semiconductor QDs have been drawing great attention recently as a material for solar energy conversion due to their high absorption coefficient, quantum confinement (tunable band gap), and MEG effects. QDSCs are burgeoning semiconductor QD solar cells that show promising developments for the next generation of solar cells. Future works should focus on improving the performance of the solar cells as follows: 1) designing new semiconductor QDs with a large wavelength range of optical absorption in terms of quantum confinement; 2) getting MEG effect enhancement of QDs by reducing the threshold energy; and 3) constructing suitable porosity for photoelectrodes to load more QDs and decrease the charge recombination. However, QDSCs are still in their infancy and face huge challenges in their development. With the recent advances in the study of semiconductor QDs, we expect major breakthroughs in developing QDSCs in the future.
